# Hormonal Treatment and Pelviscopic Myomectomy

**DOI:** 10.1155/DTE.1.217

**Published:** 1995

**Authors:** L. Mettler, E. Alvarez-Rodas, K. Semm

**Affiliations:** 1 Department of Obstetrics and Gynecology University of Kiel Michaelis-Midwifery School Germany; 2 Michaelisstr. 16 Kiel 24105 Germany

## Abstract

In cases of benign lesions, pelviscopy is used in about 70% of all abdominal operations at our Department of Obstetrics and Gynecology. From 1990 to 1992, 851 patients with myomas were treated by surgery. In 57% pelviscopy, in 2% laparotomy, and in 1% hysteroscopic myomectomies were treated. In 11%, a CISH (Classical Intrafascial SEMM—serrated edged macro morcellator—Hysterectomy) without colpotomy was applied using the operative technique of pelviscopy or laparotomy. The application of this new surgical technique preserves the patient's pelvic floor (diaphragm pelvis and urogenitalis), its blood supply, and neural function. Details of the surgical techniques used in pelviscopic myomaenucleations are described. One hundred sixteen patients were treated with a gonadotropin releasing hormone analogue (GnRH-a) before the pelviscopic myomaenucleation took place. In this study, 64 (55%) patients received 3,75 mg leuprorelin, and 52 (45%), patients 3.75 mg triptorelin. The monthly injections took place over a period of 3 to 6 months. After 3 months, an identical reduction of the myomas of about 10% to 50% was observed in 103 patients (88%) in both therapy groups. The preservation of the uterus by this minimal invasive surgery technique was generally accepted. No serious complications occurred.


*Diagnostic and Therapeutic Endoscopy*, 1995, Vol. 1, pp. 217–221
Reprints available directly from the publisher
Photocopying permitted by license only

© 1995 Harwood Academic Publishers GmbH
Printed in Singapore

### Hormonal Treatment and Pelviscopic Myomectomy

L. METTLER, E. ALVAREZ-RODAS, and K. SEMM

*Department of Obstetrics and Gynecology, University of Kiel, and Michaelis-Midwifery School, Germany*

*(Received June 8, 1994; in final form October 31, 1994)*

In cases of benign lesions, pelviscopy is used in about 70% of all abdominal operations at our Department of Obstetrics and Gynecology. From 1990 to 1992, 851 patients with myomas were treated by surgery. In 57% pelviscopy, in 2% laparotomy, and in 1% hysteroscopic myomectomies were treated. In 11%, a CISH (Classical Intrafascial SEMM—serrated edged macro morcellator—Hysterectomy) without colpotomy was applied using the operative technique of pelviscopy or laparotomy. The application of this new surgical technique preserves the patient's pelvic floor (diaphragm pelvis and urogenitalis), its blood supply, and neural function. Details of the surgical techniques used in pelviscopic myomaenucleations are described. One hundred sixteen patients were treated with a gonadotropin releasing hormone analogue (GnRH-a) before the pelviscopic myomaenucleation took place. In this study, 64 (55%) patients received 3,75 mg leuprorelin, and 52 (45%), patients 3.75 mg triptorelin. The monthly injections took place over a period of 3 to 6 months. After 3 months, an identical reduction of the myomas of about 10% to 50% was observed in 103 patients (88%) in both therapy groups. The preservation of the uterus by this minimal invasive surgery technique was generally accepted. No serious complications occurred.

**KEY WORDS:** Gonadotropin releasing hormone analogue, laparoscopy, leiomyoma, pelviscopy, uterine fibroids, minimally invasive organ—preserving surgery

### INTRODUCTION

Uterine myomas are the most common tumors of the female genital tract. Hysterectomy is the typical therapy in patients whose families are complete. In the last decade, operative pelviscopy had a breakthrough in the practice of gynecology. Recent progress has been made with the development of appropriate instrumentation. Myomectomy appears to be a safe technique with the advantages of pelviscopic surgery. For the preservation of the uterus, especially for those infertile women desiring organ-preservation, minimal invasive surgery is the current accepted operative ideal method (1–4).

Furthermore, GnRH-a can effect a shrinkage of leiomyoma before a myomectomy. GnRH-a preoperative therapy offers an alternative approach to hysterectomy in patients with the multiple uterine myomas. This therapy induces a reversible hypogonadotropic hypogonadal environment. During gonadotropin-analogue therapy the women are amenorrheic (5–8).

It is the aim of this paper to summarize our experience with organ-preserving pelviscopic operative techniques for the uterus and to evaluate this procedure as performed in our Department of Obstetrics and Gynecology in Kiel.

### MATERIAL AND METHODS

### Patients

In nearly 500 patients between January 1990 and December 1992 myomectomies were performed by pelviscopy and laparotomy. The patient groups were subdivided according to age and parity. The numbers of myomas were estimated by vaginal ultrasound and by pelviscopy. Only the anterior-posterior diameters were measured in vaginal ultrasound (Siemens, Sonoline SL-1). The location of the myomas was described, and according to the classical division they were further subdivided into subserous, intramural, and submucous myomas. Additional indications for the myoma excision were sterility, pain and pressure symptoms, bleeding abnormalities, prophylactic myomectomy, and others. Histologically we checked for leiomyomas, adenoleiomyomas, and leiomyosarcomas. One hundred sixteen patients with pelviscopic myomectomy were pre-

Address for correspondence: L. Mettler M.D., Michaelisstr. 16, 24105 Kiel, Federal Republic of Germany.

treated for 3 or 6 months with GnRH-a. We compared leuprorelin (Enantone Gyn®) and triptorelin (Decapeptyl Depot®). The anterior-posterior diameter of myomas was measured on vaginal ultrasound scan before and after GnRH—a therapy.

### Technique

### *Pedunculated Myomas*

These myomas were resected after endocoagulation of the pedicle or after the placement of loops or staplers. Technique for subserous myomas included:

1. Injection in the walls with a 0.05% POR 8®*solution
2. Positioning of a Roeder-Loop around the base of the myoma
3. Endocoagulation of the capsule
4. Incision of the capsule
5. Myoma enucleation
6. Continuous tension on the Roeder-Loop
7. Dissection of the myoma
8. Closure of the capsule with the Roeder-Loop

### *Intramural myomas*

The excision of these myomas up to 500 to 700 g is possible. They may interfere with embryo growth in pregnancy, produce uterine bleedings, and certainly give way to hysterectomy, because of increasing pathologic symptoms such as pressure on the urinary bladder or rectum. Therefore an early excision of myoma is advisable. The necessary endoscopic surgical steps are the following:

1. Injection of two to three dosages of 10 ml of a POR 8®* 0.05% solution.
2. Endocoagulation patch with the point coagulator at 100/120°C.
3. Incision with the micro- or macro scissors.
4. Myoma enucleation with the myoma enucleator.
5. Final enucleation and dislocation from the uterus.
6. Endocoagulation.
7. Suture of the wound with an endosuture and extracorporeal knotting technique. Usually 2–4 sutures in one layer going deep through the myometrium.
8. Myoma extraction with the morcellator.

Even myoma lying in the depth of the uterine muscle visible by ultrasonography and not by pelviscopic inspection can be excised. If they compress the uterine cavity, they can be an obstacle for implantation and should be resected pelviscopically or via hysteroscopy.

### *Submucous myomas*

In liquid hysteroscopy such myomas can be resected by laser or by electrocoagulation (10). (*) POR 8® Is a vasopressin derivative from Sandoz.

### RESULTS

Of 851 surgical procedures (as myomectomies; total abdominal and vaginal hysterectomies, and CISH procedures over a period of 3 years) performed for myomatous lesions of the uterus, 482 (57%) were pelviscopic myomectomies. In only 18 cases was a myomectomy via laparotomy performed because of very large or multiple myomas; in 8 cases a hysteroscopic or direct vaginal myomectomy was carried out (Table 1, Fig. 1). Pelv-CISH means pelviscopic classical intrafascial SEMM hysterectomy, Lap-CISH means laparotomic classical intrafascial SEMM hysterectomy (11).

The average age of the patients was 43.2 years (range, 21 to 69 years). The parity reveals that 60% of the cases are nulliparous, and only 3.7% women had given birth to more than 2 children. Even in perimenopausal women with myomas and pressure symptoms we believe endoscopic myomectomy to be indicated.

We found infertility in 50% of the cases, pain in 18%, and bleeding abnormalities in 16% as indication for the endoscopic myomectomy (Fig. 2). In 16%, fibroids were detected, but they did not present the indication for this intervention. In 275 cases (57%), myomectomy was the only procedure performed. In the 207 cases, other procedures also were performed such as tuboplasty, endocoagulation of endometriosis, ovarian cystectomy, and adhesiolysis. We are a reference center for infertility patients, and we believe in myomaexcision at an early stage of myoma development.

The combined evaluation of the vaginal and the abdominal ultrasound data and the pelviscopic inspection states the number of all myomas and size of the largest myoma. In 70% of the cases 1 or 2 myomas were excised and in 30%, 3 to 6 myomas were excised. Whereas 36% of all myomas were localized on the uterus fundus, 39% were found on the posterior wall of the uterus. Multiple locations occurred in 26% (Fig. 3). According to their penetration depth, nearly 50% of all myomas were subserous.

**Table 1** Spectrum of Treatment of Uterus Myomatosus Patients

| *1990 to 1992, UFK KIEL N = 851 Patients (100%)* | |
|---|---|
| Pelviscopic Myomenucleation | 482 (57%) |
| Laparotomic Myomenucleation | 18 (2.0%) |
| Hysterectomy | |
| Pelvic CISH* | 57 (6.7%) |
| Lap. CISH | 37 (4.0%) |
| Abdominal | 194 (23%) |
| Vaginal | 55 (6.4%) |
| Hysteroscopic Myomenucleation | 8 (1.0%) |

*CISH indicates classical Intrafascial SEMM (serrated edged macro morcellator) hysterectomy; pelv, by pelviscopy.

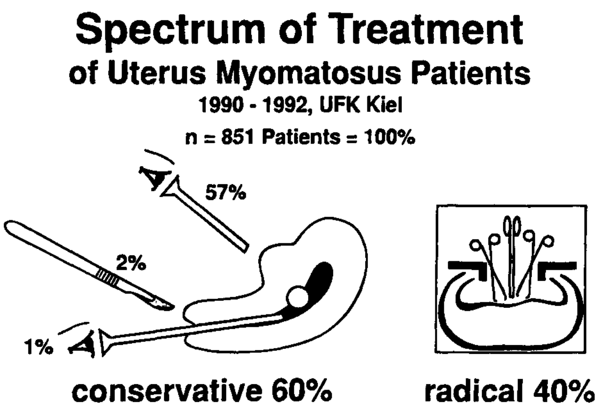

**Figure 1** Spectrum of treatment of uterus myomatous patients 1990 to 1992, UFK Kiel Pelviscopic treatment of pedunculated myomas.

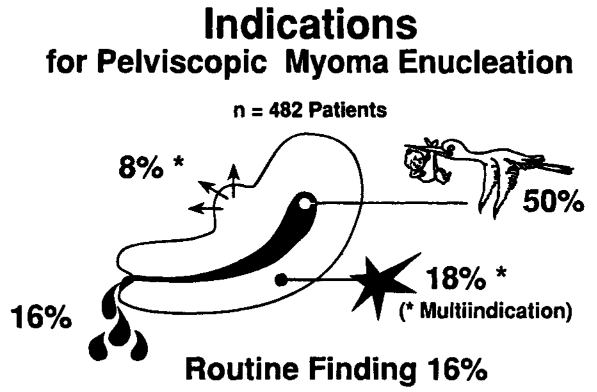

**Figure 2** Indications for pelviscopic myoma enucleation. Pelviscopic myomectomy of subserous myomas.

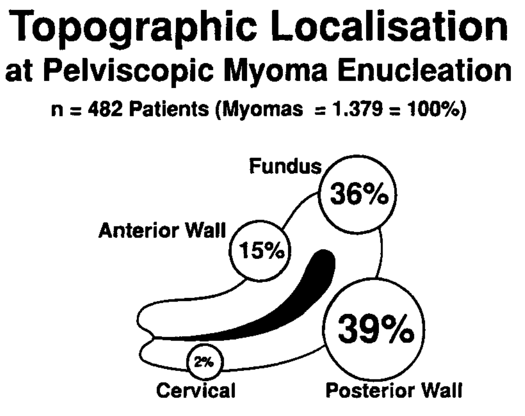

**Figure 3** Topographic localization at pelviscopic myoma enucleation. Pelviscopic myomectomy in cases of

Suture-ligature techniques were used to close the wounds in 66% of the patients and in 9%, endocoagulation after the myoma excision. In 25% of the cases both techniques were applied. One thousand three hundred seventy nine myomas were removed in 482 patients. While 670 myomas had a mean diameter between 10 mm and 39.9 mm (48.6%), 709 had a mean diameter between 40 mm and 110 mm (51.4%) in vaginal ultrasound. The histology shows mainly leiomyomas and adenoleiomyomas (Table 2). In one case, in 1992, a leiomyosarcoma unexpectedly was found on histology. After hysterectomy no additional leiomyosarcoma was detected in the uterine specimen. The analysis of both GnRH-a pretreated patient groups (the 3-month treatment group compared with the 6-month treatment group), revealed that after 3 months the treatment had already reached maximum effect. Comparing leuprorelin and triptorelin acetate, the myoma reduction was finally 10% to 50% in both groups (Tables 3, 4 and 5). The average blood loss was < 200 mL, no transfusions were necessary. Postoperative bleeding occurred in 5 of 482 patients and was stopped by a second-look pelviscopy. No laparotomy was necessary.

### DISCUSSION

In Kiel, operative pelviscopy has replaced 70% of the classic gynecological laparotomies (9). The goal of minimal invasive surgery is to preserve organs. Postoperative sequels such as adhesions, intestinal obstruction, chronic abdominal pain, etc. are thus decreased (9, 11) and myomectomy seems to be a safe technique with the advantages of pelviscopic surgery. The postoperative recovery time is much shorter and more comfortable, and postoperative pain is reduced, making it possible for patients to eat on the same day and get up in the evening. We have seen that pretreatment with GnRH-a is desirable in cases of myomas to be resected pelviscopically. Analyzing the time taken for the myoma reduction, the response can be predicted in most cases as early as 8 weeks after the first injection (2). The gonadotropin-re-

**Table 2** Histological Pathomorphology

*Pelviscopic Myoma Enucleations at the UFK Kiel between January 1990 to December 1992*
*N = 482 Patients*

| *Size* | *1 < 4 cm* | *4 to 10 cm* | *Total* |
|---|---|---|---|
| Histology | | | |
| Leiomyoma | 304 | 207 | 511 |
| Regressive leiomyoma | 267 | 304 | 571 |
| Necrotising leiomyoma | 4 | 56 | 60 |
| Adenoleiomyoma | 32 | 33 | 65 |
| Cellular leiomyoma | 63 | 108 | 171 |
| Sarcomas | 0 | 1 | 1 |
| Total | 670 | 709 | 1379 |

**Table 3** Reduction of myomas

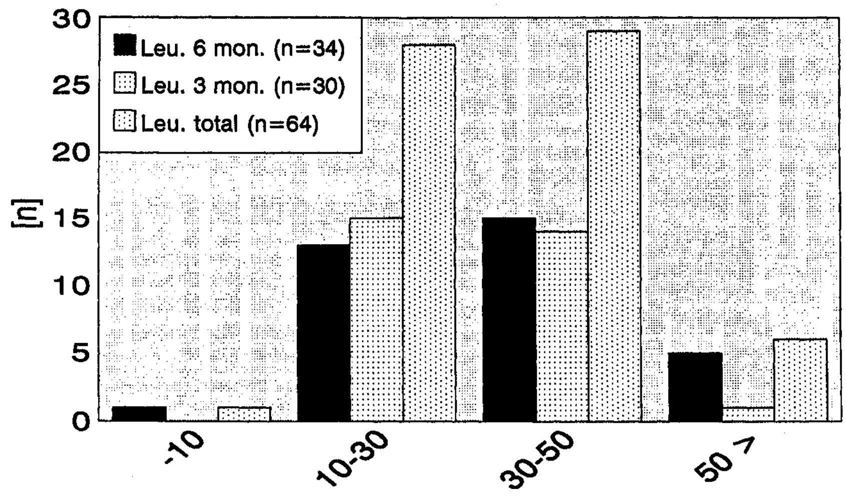

**Percent reduction in fibroid volume**

| | < 10 % | 10 - 30 % | 10 - 50 % | > 50 % |
|---|---|---|---|---|
| Leuprorelin 6 mon. (n=34) | 1 (3 %) | 13 (38 %) | 15 (44%) | 5 (15 %) |
| Leuprorelin 3 mon. (n=30) | 0 | 15 (50%) | 14 (47 %) | 1 (3 %) |
| Leuprorelin total (n=64) | 1 (2 %) | 28 (44 %) | 29 (45 %) | 6 (9 %) |

**Table 4** Reduction of myomas

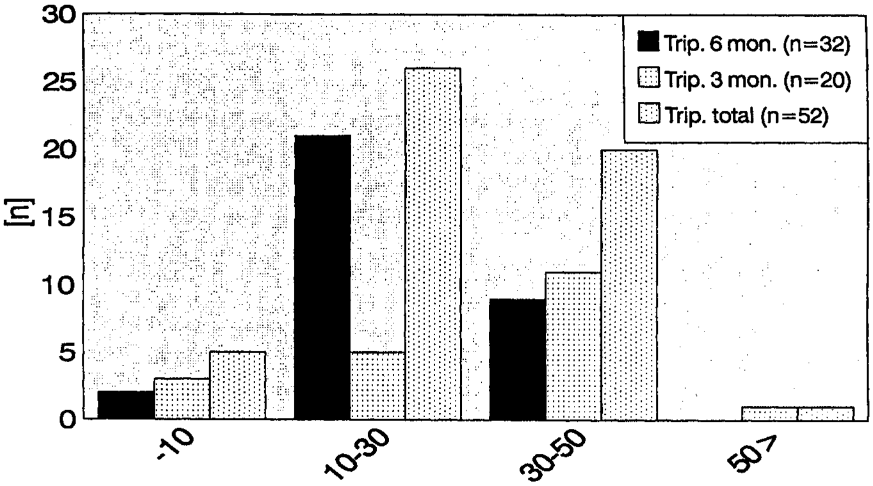

**Percent reduction in fibroid volume**

| | < 10 % | 10 - 30 % | 10 - 50 % | > 50 % |
|---|---|---|---|---|
| triptorelin 6 mon. (n=32) | 2 (6 %) | 21 (66 %) | 9 (28 %) | 0 |
| triptorelin 3 mon. (n=20) | 3 (15 %) | 5 (25 %) | 11 (55 % | 1 (5 %) |
| triptorelin total (n=52) | 5 (10 % | 26 (50 %) | 20 (38 %) | 1 (2 %) |

**Table 5** Hormonel pretreatment

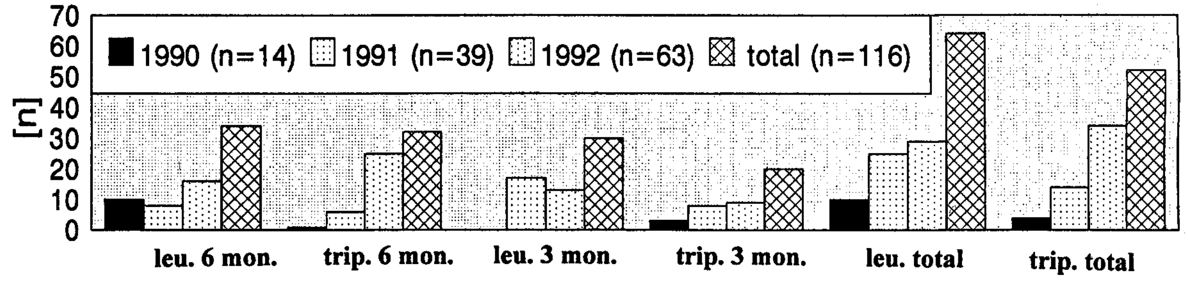

| | | | | | | |
|---|---|---|---|---|---|---|
| 1990 (n=14) | 10 (71.4%) | 1 (7.1%) | 0 (0 %) | 3 (21.4 %) | 10 (71.4 %) | 4 (28.6 %) |
| 1991 (n=39) | 8 (20.5%) | 6 (15.4%) | 17 (43.6%) | 8 (20.5 %) | 25 (64 %) | 14 (36 %) |
| 1992 (n=63) | 16 (25 %) | 25 (40%) | 13 (21%) | 9 (14 %) | 29 (46 %) | 34 (54 %) |
| total (n=116) | 34 (29 %) | 32 (28 %) | 30 (26 %) | 20 (17 %) | 64 (55 %) | 52 (45 %) |

leasing hormone analogues have potential benefits as presurgical medication in the management of uterine leimyomas; we conclude that triptorelin and leuprorelin acetates have the similar effect. The decrease in myoma volume induced by the analogue allowed a more limited intervention and prevented excessive blood loss (12, 14).

Our experience leads to the conclusion that surgical pelviscopy can be used for management of more complicated problems and can be regarded as an alternative to laparotomy. Preoperative evaluation is crucial in determining operative strategy, according to the size, number, and localization of myomas. In our experience, hysteroscopy should be systematically performed before pelviscopy, because it allows the surgeon to differentiate deep intramural myomas that should be treated by pelviscopy from submucous myomas that can be treated by hysteroscopy. Frequently submucous myomas are associated with subserous or intramural myomas. In these cases, ablation of the submucosal myoma has to be performed during the same operation by operative hysteroscopy. Our experience has shown that the mean operating time is less than 100 minutes. No serious complications occurred in 482 patients. In 5 cases, because of extensive bleeding and decrease in haemoglobin, a repeat pelviscopy had to be performed using endocoagulation and endosutures for hemostasis. After intramural or submucosal myomectomy, closure of the myometrium by sutures is recommended to prevent postoperative bleedings. A postoperative evaluation of the formation of adhesion, fertility, and normal reproductive function is being performed, and so far no case of uterine rupture after myomectomy has occurred.

### REFERENCES

1. Palmer R. Safety in laparoscopy. J Reprod Med, 1974;13:1–5.
2. Semm K. Operationslehre für endoskopische Abdominal—Chirugie FK, Schattauer, Stuttgart, 1984.
3. Semm K. Operative manual for endoscopy abdominal surgery. Friederich ER (trans., ed.), Year Book Medical Publishers, 1987.
4. Semm K, Mettler L. Technical progress in pelvic surgery via operative laparoscopy. Am J Obstet Gynecol, 1980;138:121.
5. Mettler L, Steinmuller H, Schachner-Wunschmann E. Experience with a Depot GnRH—agonist. (Zoladex) in the treatment of genital endometriosis. Hum Reprod 1991;6(5):694–698.
6. Adamson GD. Treatment of uterine fibroids: current findings with gonadotropin—releasing hormone agonists. Am J Obstet Gynecol 1992;166(2):756–61.
7. Cirkel U, Ochs H, Schneider H, Mettler L, et al. Experience with leuprorelin acetate depot in the treatment of fibroids: a German multicentre study. Clin Ther 1992;14 suppl A:37–50.
8. Van-Leusden HA. Symptom-free interval after triptorelin treatment of uterine fibroids: long-term results. Gynecol Endocrinol 1992;6(9):189–198.
9. Mettler L, Semm K. Pelviscopic uterine surgery. Surg Endosc 1992;6:23–31.
10. Hamou JE. Microhysteroscopic, une nouvelle technique en endoscopic, ses applications. Acta Endosc 1980;10:415–422.
11. Semm K. Hysterectomy via laparotomy or pelviscopy a new CASH method without colpotomy. Geburtshilfe Frauenheilkd 1991;51(12):996–1003.
12. D'Addato F, Repinto A, Andreoli C. Presurgical treatment of uterine myomas with LH-RH agonists. Clin Exp Obstet Gynecol 1992;19(1):45–50.
13. Wieacker P, Geisthovel F, Adelberger V, Breckwold M. GnRH: analogs in the therapy of uterine myomatosis. Ther Umsch 1990;47(12):951–957.
14. Friedman AJ, Lobel SM, Rein MS, Barbieri RL. Efficacy and safety considerations in women with uterine leiomyomas treated with gonadotropin releasing hormone agonists: the estrogen threshold hypothesis. Am J Obstet Gynecol 1990;163(4):1114–9.

